# Proteolytic Activity of *Bacillus subtilis* upon κ-Casein Undermines Its “Caries-Safe” Effect

**DOI:** 10.3390/microorganisms8020221

**Published:** 2020-02-06

**Authors:** Danielle Duanis-Assaf, Eli Kenan, Ronit Sionov, Doron Steinberg, Moshe Shemesh

**Affiliations:** 1 Department of Food Sciences, Institute for Postharvest Technology and Food Sciences, Agricultural Research Organization (ARO), Volcani Center, Rishon LeZion 7505101, Israel; danielle.assaf@mail.huji.ac.il; 2 Biofilm Research Laboratory, Institute of Dental Sciences, Faculty of Dental Medicine, Hebrew University-Hadassah, Jerusalem 9112001, Israel; Elikemail@gmail.com (E.K.); Ronitsionov@gmail.com (R.S.); dorons@ekmd.huji.ac.il (D.S.)

**Keywords:** *Bacillus subtilis*, *Streptococcus mutans*, biofilm formation, milk caseins

## Abstract

Milk is believed to be a relatively “caries-safe” food. This belief relies on the fact that caseins, which constitute around 80% of milk’s protein content, were found to inhibit the adhesion of *Streptococcus mutans* to enamel and, therefore, decrease biofilm formation. While *S. mutans* is considered a leading cause of dental disorders, *Bacillus subtilis* is a non-pathogenic foodborne bacterium, frequently contaminating milk and its products. This study aimed to investigate the effects of dairy-associated foodborne bacteria such as *B. subtilis* on biofilm formation by *S. mutans* in the presence of casein proteins. Our results indicate that there is a significant decrease in total biofilm formation by *S. mutans* exposed to a casein protein mixture in a mono-species culture, whereas, in the co-culture with *B. subtilis*, an inhibitory effect of the caseins mixture on *S. mutans* biofilm formation was observed. Proteolytic activity analysis suggested that *B. subtilis* is capable of breaking down milk proteins, especially κ-casein, which enables biofilm formation by *S. mutans* in the presence of milk caseins. Therefore, these findings may challenge the assumption that milk is “caries-safe”, especially in a complex microbial environment.

## 1. Introduction

Milk is a complex colloidal mixture of proteins, fats, sugars, and minerals, with some in suspension and some in solution [1]. Essentially, since milk contains all the necessary components required for the development and maintenance of the mammalian organism, a dairy-rich diet is indispensable in most industrial nations [2,3]. Milk provides all the necessary energy and nutrients to ensure proper growth and development; it is crucial with respect to bone mass formation, as well as the prevention of several chronic conditions such as cardiovascular diseases, some forms of cancer, obesity, and diabetes [4]. Moreover, for many years, milk and its products were also considered “caries-safe” products [3]. 

Caries is the most common chronic infectious disease related to the oral cavity [5]. Biofilm formation represents the first and foremost step in caries development and progression [6]. Different sophisticated mechanisms of adherence account for the distribution of different species to a particular habitat in the oral tissues [7]. In addition to the adherence properties, dental biofilm displays a complex structure due to the extracellular matrix, which contributes to its resistance to mechanical removal, constitutes a reservoir for nutrients and bacteria, and determines the interactions of the biofilm with the environment [5]. 

Considered as the primary causative bacterium of dental caries, *Streptococcus mutans* owes its virulence to its capability to form robust biofilms on dental surfaces [8]. The bacterial adhesion to the surface, which is highly related to its cariogenic activity, is mediated by the synthesis of extracellular polysaccharides (EPS) by the extracellular enzymes glucosyltransferase (GTF) and fructosyltransferase (FTF) [9,10]. Accumulation of *S. mutans* and other oral bacteria as a biofilm is the result of the bacteria’s self-adhesion mechanisms, but it is also highly dependent on dietary components [6]. Furthermore, consequently to the access of nutrients, organic acids are generated by the bacterial fermentation, which result in the acidification of the environment and provide a main threat for the enamel integrity [11]. Thus, caries is highly dependent on dietary components, which can influence bacterial adhesion and biofilm formation [6].

Previous studies indicated the possible effect of milk and/or its products on the cariogenicity potential of *S. mutans* [1]. While lactose enhances biofilm formation and acid production by *S. mutans* [3,12], other milk components might have an anti-biofilm and/or anticariogenic effect [13,14,15]. It was suggested that milk proteins, for instance, κ-casein and immunoglobulin G, inhibit dental biofilm formation [16]; thus, milk might act as a buffer against acid production [17]. Moreover, κ-casein may decrease the biofilm formation by attaching to the adhesion-like protein and inhibiting the ability of bacteria to attach to surfaces [13,14,15,18]. Furthermore, the anti-biofilm effect could be reached by interacting with the GTF enzyme and reducing its activity [15]. Moreover, caseins may stabilize Ca-PO_4_ molecules (ACP) and contribute to the re-mineralization of the enamel [19]. 

However, none of these studies tested the possible role of foodborne bacteria on the cariogenicity potential of milk components, including milk proteins. In the present study, we chose the foodborne bacterium *Bacillus subtilis* as a model microorganism for milk-associated bacteria. *B. subtilis* is a non-pathogenic, spore-forming bacterium. Indeed, *Bacillus* species are found to be predominant in both raw and pasteurized milk. Moreover, a recent study demonstrated that *B. subtilis* and *S. mutans* are capable of forming a dual-species biofilm [20]. Therefore, it was of interest to test their possible role in the dynamics between cariogenic *Streptococci* and the milk components [18]. It is also hypothesized that a possible production of the proteolytic enzymes, enabling the metabolization of major milk components, by *Bacillus* would affect the cariogenicity potential of *S. mutans* [21]. Therefore, the aim of this study was to investigate the role of a milk-associated bacterium *Bacillus subtilis* in the cariogenecity potential of *S. mutans.*

## 2. Materials and Methods

### 2.1. Bacterial Strains and Growth Media

Cultures of *S. mutans* strain UA159 were grown overnight in brain heart infusion (BHI) broth (Acumedia, Lansing, MI) at 37 °C in 95% air/5% CO_2_. *B. subtilis* wild-type (WT) strain NCIB3610 was routinely maintained in Lysogeny broth (LB, Neogen, Lansing, MI). To generate starter cultures, one colony of *B. subtilis* from a fresh LB agar plate was grown as a suspension in LB via incubation at 37 °C/150 rpm for 5 h. All experiments were conducted using bacterial cells in the late exponential phase.

### 2.2. Lactose Preparation

A stock 50% lactose (J.T. Baker, London, United Kingdom) solution was prepared in distilled deionized water (DDW) and sterilized using a 0.2-µm filter (Whatman, Dassel, Germany). The stock solution of lactose was diluted in BHI to final concentrations of 3% *w*/*v*.

### 2.3. Casein Preparation

A stock of 4% *w*/*v* casein (Sigma Aldrich, St. Louis, MO, USA) solution was dissolved in double-distilled water (DDW) pH 7.4 with 15-min sonication. The solution was sterilized in an autoclave for 10 min at 121 °C. The stock solution was diluted in 2× BHI to obtain 2% *w*/*v* casein. Dilutions for work solutions of 1.5%, 1%, or 0.5% casein were conducted in BHI [22].

### 2.4. Mono- and Dual-Species Biofilm 

For mono-species biofilm, overnight cultured *S. mutans* (optical density (OD_600 nm_) ≈ 1) was introduced (by 1:10 ratio) into the fresh BHI supplemented with 3% lactose with or without addition of various concentrations of casein in a 96-well plate. The plate was incubated at 37 °C in 95% air/5% CO_2_ for 24 h. 

For *B. subtilis* mono-species biofilm, a starter culture was introduced (in the ratio 1:100) into the fresh BHI supplemented with 3% lactose with or without addition of various concentrations of casein in a 96-well plate. The plate was incubated at 37 °C in 95% air/5% CO_2_ for 24 h. 

For the dual-species biofilm, starter cultures of *S. mutans* (approximately 2.5 × 10^7^ colony-forming units (CFU)) and *B. subtilis* (around 2.5 × 10^5^ CFU), generated as described above, were added to BHI supplemented with 3% lactose with or without addition of various concentrations of casein in a 96-well plate. The plate was incubated at 37 °C in 95% air/5% CO_2_ for 24 h. The ratio of *B. subtilis* to *S. mutans* cells seeded to obtain the dual-species biofilm was approximately 1:100 [20]. 

### 2.5. Characterization of Biofilm Structure by Confocal Laser Scanning Microscopy (CLSM)

CLSM was used for visualization of deeper layers of the biofilm and quantification of the bacterial cells in the biofilm. The biofilms were prepared as described above. To examine the vitality of the biofilms, LIVE/DEAD BacLight fluorescent dye (Life Technologies, Carlsbad, CA, USA) was added to the washed biofilms for 20 min to facilitate differentiation between the live and dead *S. mutans* cells.

To avoid proteolytic activity, a protease inhibitor cocktail (Bio-rad, Hercules, CA, USA), was added to the media according to the manufacturer’s recommendations. 

SYTO 9 fluorescence was measured using 488-nm excitation and 515-nm emission filters, and propidium iodide (PI) fluorescence was measured using 543-nm excitation and 570-nm emission filters. The biofilm depth was examined by generating the optical sections that were acquired at spacing steps of 10 µm.

The generated biofilms were visualized using a Zeiss LSM510 CLS microscope (Carl Zeiss, Oberkochen, Germany). Three-dimensional images were constructed using Zen software (Carl Zeiss). The biofilm biomass and live/dead ratio were calculated as green and red fluorescence intensity using ImageJ software (http://rsb.info.nih.gov/ij). 

### 2.6. Quantification of Biofilm Biomass using DNA Quantification

The mono- and dual-species biofilms were grown as described above on a sterile coverslip (5 mm diameter, glass, Barnaor, Israel). The coverslips were removed and washed twice with PBS and placed in a new 48-well plate. 

Extraction and quantification of DNA were performed as described previously [23]. Briefly, 320 µL of NaOH (0.05 M) (Bio Lab Ltd., Jerusalem, Israel) and 80 µL of water (DEPC treated) (Bio Basic Canada Inc, Markham, Ontario, Canada) were added to each well. The plate was immersed in a water bath at 60 °C for 1 h, and 37 µL of Tris buffer (pH 7.0) (Eastman Kodak Company, Rochester, NY, USA) was added to each well after the heating period. The extracted DNA samples were stored at −20 °C until use.

The DNA samples from the different biofilms were quantified using a quantitative PCR reaction with specific primers for 16S ribosomal RNA (rRNA), either for *S. mutans* or *B. subtilis*, using the ABI prism instrument (Applied Biosystems Prism 7300, Foster City, CA, USA). The amount of DNA was quantified according to the specific standard curve. The bacterial genomic DNA for the standard curve analysis was extracted from starter cultures of *S. mutans* UA159 or *B. subtilis* NCIB3610 using a GenElute Bacterial genomic DNA kit (Sigma Aldrich, St. Louis, Missouri, USA) according to the manufacturer’s instructions. The genomic DNA was stored at −20 °C.

### 2.7. Biofilm Visualization by Scanning Electron Microscopy (SEM)

For biofilm visualization, hydroxyapatite (HA) discs (5 mm diameter, 2 mm thickness, Clarkson chromatography products inc., PA. USA) were insert into a 48-well plate. The biofilm formation was conducted as described above. After 24 h, the HA discs were taken and washed twice using DDW. All samples were fixed using 4% formaldehyde for 20 min and washed using DDW. The samples were gold-coated and visualized using an analytical Quanta 200 Environmental High-Resolution Scanning Electron Microscope (EHRSEM) (FEI, Eindhoven, The Netherlands) [24]. 

### 2.8. Proteolytic Activity Assay 

*S. mutans* and *B. subtilis* starters were seeded on skim milk supplemented with 1.5% agar. The samples were incubated at 37 °C for 24 h. Proteolytic activity was determined according to the change in the color of skim milk (from white to transparent) around bacterial colonies [25].

### 2.9. Casein Breakdown in Solution by B. subtilis and S. mutans Lysate

Starter cultures of *S. mutans* and *B. subtilis* were grown in LB or BHI (respectively) for 14 h. After centrifugation (5000× *g*, 15 min, 4 °C), the bacteria were washed twice in 20 mL of sodium acetate buffer 2 M (*w*/*v*), pH 7.2, containing 0.04% (*w*/*v*) NaN_3_. Finally, the bacteria were resuspended in 6 mL of sodium acetate buffer. The cells were disrupted with MP-cell disrupter: 1 min, 60 s, 5 m/sec in 1.5-mL tubes containing acid-washed glass beads. The suspension was then centrifuged (12,000× *g*, 5 min, RT), and the supernatant containing enzymes was incubated on ice. The lysates were mixed 1:1 with 5 mg/mL casein solution or κ-casein for 2 h at 37 °C, 150 rpm. After sampling, 24 µL of sample buffer (4×) and 9 µL of 1,4-dithiothreitol (DTT) 0.25 M were added to the sample. The proteases of the lysates were inactivated in each sample by heating at 90 °C for 15 min. The samples were taken to analysis on 15% SDS-PAGE gel. The gels were stained using silver stain according to the manufacturer’s protocol [26]. 

### 2.10. Casein and κ-Casein Zymography

Casein and κ-casein zymograms were carried out as described previously with slight modification [27]. The supernatant of overnight cultures of *B. subtilis* and *S. mutans* was diluted 1:1 with zymogram sample buffer (0.5 M Tris-HCl, pH 6.8, 10% SDS, 20% glycerol, and 0.5% bromophenol blue). The electrophoresis was performed in an ice bath at a constant current of 20 mA. 

### 2.11. Statistical Analysis

The data were statistically analyzed using the *t*-test. When needed, ANOVA, followed by a post hoc *t*-test with Bonferroni correction, was conducted. All applied tests were two-tailed, and *p* < 0.05 was considered statistically significant.

## 3. Results

### 3.1. B. subtilis Enables Biofilm Formation by S. mutans in the Presence of Caseins

**Figure 1:**
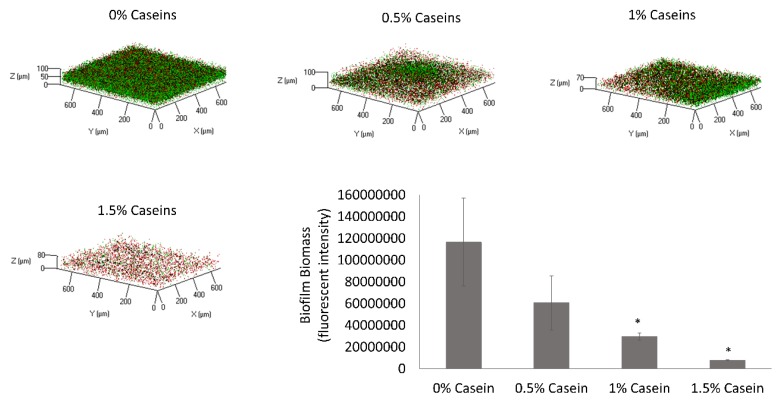
Casein proteins decrease biofilm formation by *Streptococcus mutans*. *S. mutans* cells were grown in brain heart infusion (BHI) supplemented with 3% lactose with various concentrations of the casein proteins mixture (0%, 0.5%, 1%, or 1.5%). After 24 h of incubation at 37 °C in 95% air/5% CO_2_, the formed biofilms were washed twice. The biofilms were stained with Syto 9 (green) labeling live bacteria and propidium iodide (PI) (red) labeling dead bacteria. The graph displays data of fluorescent integrity of the formed biofilms as calculated by the ImageJ software. The pictures are representative of four biological repeats, each preformed in duplicate. * *p* < 0.05 compared to control (0% casein).

The first step in this study was to determine the effect of the major milk caseins (αs1, αs2, β, and κ at the same ratio as they appear in bovine milk) on the surface attachment and biofilm formation by *S. mutans*. This was investigated using CLSM in the presence of various doses of the casein proteins in suspension. The cell-specific fluorescent intensity was quantified, which represents the amount of biofilm biomass. It was found that the biofilm biomass was reduced by 30% following the addition of casein protein mixture at a concentration of 0.5%, with a decrease of up to 94% with the addition of 1.5% casein. Moreover, the inhibition of biofilm formation increased significantly with the increase in casein fraction in the media in a dose-dependent manner (Figure 1). According to the microscopy observation, the inhibitory effect of the casein mixture resulted in a notable depletion of live cells that were adhered to the surface. Importantly, the decrease in the biofilm formation was not a result of growth inhibition based on the bacterial growth curve analysis (Figure S1).

**Figure 2:**
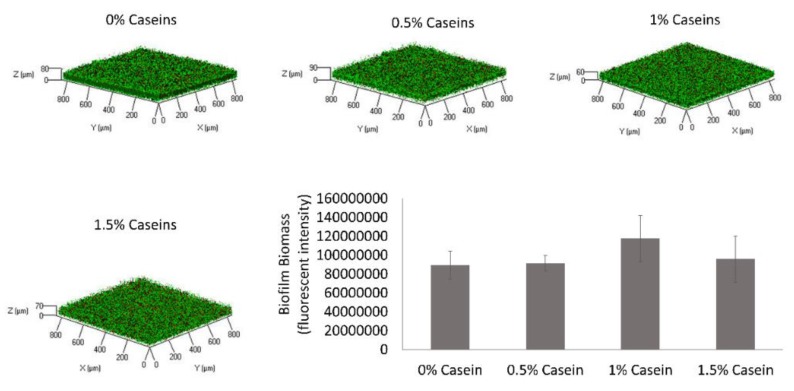
*Bacillus subtilis* enables biofilm formation by *Streptococcus mutans* in the presence of casein proteins. *S. mutans* and *B. subtilis* cells were grown in BHI supplemented with 3% lactose with various concentrations of the casein proteins (0%, 0.5%, 1%, or 1.5%). After 24 h of incubation at 37 °C in 95% air/5% CO_2_, the formed biofilms were washed twice. The biofilms were stained by Syto 9 (green) labeling live bacteria and PI (red) labeling dead bacteria. The graph displays data of fluorescent integrity of the formed biofilms as calculated by the ImageJ software. The pictures are representative of four biological repeats, each performed in duplicate. * *p* < 0.05 compared to control (0% casein).

It was further hypothesized that certain milk-associated bacteria, for instance, *Bacillus* species, would affect the ability of milk caseins to mitigate biofilm formation by *S. mutans*. Thus, the effect of the casein protein mixture was tested in the presence of *B. subtilis*. It was found that the biofilm inhibitory effect of the milk caseins was lost in the dual-species culture of *B. subtilis* and *S. mutans* (Figure 2). This result indicates the ability of *B. subtilis* to interfere with the biofilm inhibitory processes caused by the caseins.

### 3.2. S. mutans is the Predominant Bacterium in the Dual-Species Biofilm

**Figure 3:**
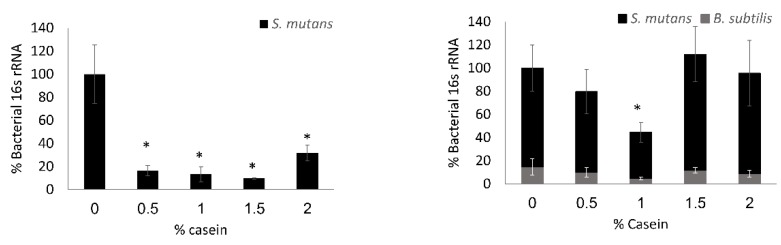
Relative composition of the species within the biofilm. *S. mutans* cells were grown with or without *B. subtilis* in BHI supplemented with 3% lactose with various concentrations of the casein proteins (0%, 0.5%, 1%, or 1.5%). After 24 h of incubation at 37 °C in 95% air/5% CO_2_, the formed biofilms were washed twice. The DNA extraction was conducted using NaOH and real-time qPCR based on the species-specific 16S ribosomal RNA (rRNA) primers to quantify the amount of each bacterium. The data display the mean ± SD of four biological repeats, each performed in triplicate. * *p* < 0.05 compared to control (0% casein).

To verify the above results and compare the bio-existence of each bacterium in the dual-species biofilm, a quantification of the 16S rRNA gene level was conducted. The quantification analysis shows a significant decrease of around 80% of DNA amount in the tested concentrations of the casein protein mixture in the *S. mutans* mono-species biofilm (Figure 3). However, there was no significant change in the amount of biofilm biomass in total (except for 1% casein) in the dual-species biofilms (Figure 3). Moreover, the qPCR quantification of 16S rRNA specifically indicated that the formed biofilm mainly consisted of *S. mutans* (Figure 3) and that there was no difference in the ratio between the bacterial species (Figure 3). 

### 3.3. B. subtilis Enables the S. mutans Attachment to Hydroxyapatite Discs in the Presence of Casein Proteins 

**Figure 4:**
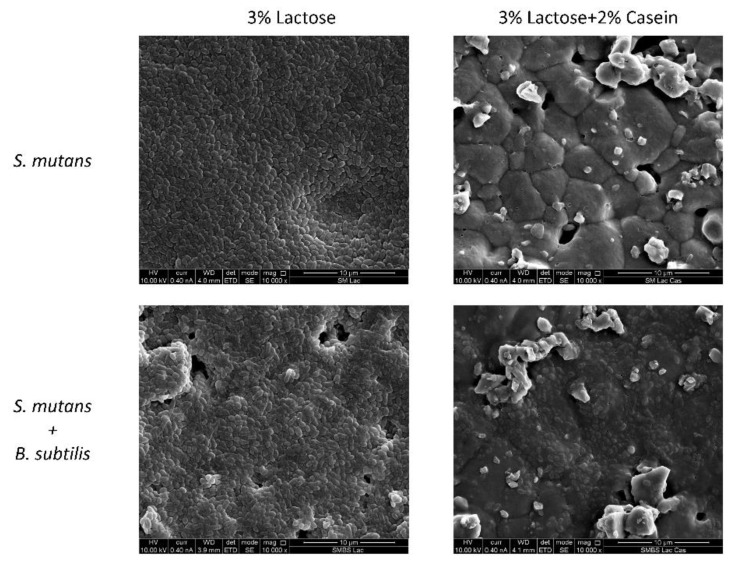
*B. subtilis* enables *S. mutans* attachment to hydroxyapatite discs in the presence of casein proteins. Either mono- or dual-species cultures of *S. mutans* were seeded on hydroxyapatite discs in BHI supplemented with 3% lactose with or without addition of the casein protein mixture (2%). After 24 h of incubation at 37 °C in 95% air/5% CO_2_, the discs were washed twice and fixed with 4% formaldehyde. The samples were taken for visualization under SEM. The pictures represent the mono-species biofilm formed by *S. mutans* (upper panel) and the dual-species biofilm of *S. mutans* and *B. subtilis* (lower panel) in the presence of 3% lactose (right) and 3% lactose with addition of the mixture (left).

To resemble conditions of the oral cavity and the tooth surface, an experiment was conducted on hydroxyapatite discs that could represent the tooth enamel (Figure 4). As expected, the cells of *S. mutans* adhered to the hydroxyapatite surface in the presence of lactose in either mono- or dual-species cultures, forming a robust biofilm (Figure 4). However, a difference between the mono- and dual-species cultures was seen in the presence of the casein mixture. Although there was no notable adherence of *S. mutans* cells onto the hydroxyapatite surface in the mono-species culture, a significant amount of *S. mutans*’ cells were found attached to the surface exposed to the dual-species culture (with *B. subtilis*) (Figure 4).

### 3.4. B. subtilis Has Proteolysis Ability on Milk Proteins

**Figure 5:**
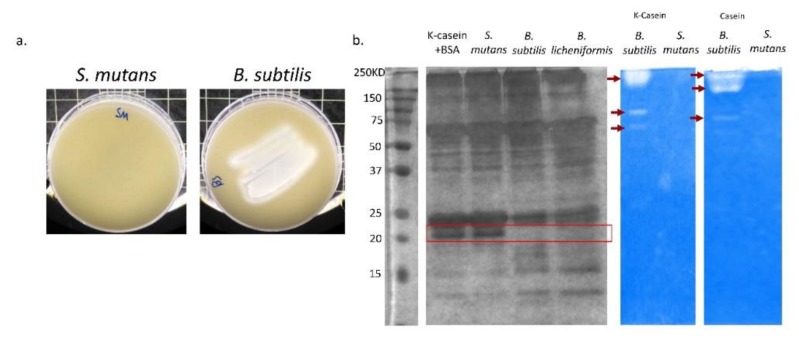
Proteolytic activity of *B. subtilis* explains a breakdown of κ-casein. (**a**) *S. mutans* or *B. subtilis* was speared on skim milk plates. After 24 h of incubation at 37 °C in 95% air/5% CO_2_, the plates were photographed. The clearance area in the *B. subtilis* plate demonstrates its ability to break down milk proteins. (**b**) On the left: SDS-PAGE of *S. mutans*, *B. subtilis* and *B. lichenifrmis* lysates after 2 h of incubation with κ-casein. In both of the *Bacillus* species, the band of κ-casein is absent, suggesting their protease activity. Bovine serum albumin (BSA) was used as internal standard for the amount of protein loaded. On the right: zymogram gel of κ-casein and total casein proteins. The supernatant of overnight cultures of *B. subtilis* and *S. mutans* was loaded on the gel. White bands indicate the enzymatic activity and hydrolysis of casein proteins (also marked by red arrows).

The inhibitory effect of milk caseins on biofilm formation by *S. mutans* is related to κ-casein and its ability to interfere with bacterial adhesion [14]. Therefore, it was hypothesized that the proteolytic activity of *B. subtilis* cells would account for disabling the κ-casein-mediated inhibition of the *S. mutans* biofilm formation. It was found that *B. subtilis* displays a strong proteolytic activity seen by clearance of around the bacterial smear on the skim-agar plates (Figure 5a). Furthermore, the hydrolysis profiles of κ-casein based on the proteolytic activity of either *S. mutans* or *B. subtilis* lysates were assessed. Dissimilarly to the *S. mutans* lysate, the proteolytic activity of the *B. subtilis* lysate resulted in the absence of the κ-casein band (Figure 5b). Moreover, the κ-casein breakdown profile was compared to that of the dairy-isolated *Bacillus licheniformis* [28], which displayed a similar phenotype (Figure 5b).

### 3.5. B. subtilis Proteolysis Ability Enables Biofilm Formation in the Presence of Caseins

**Figure 6:**
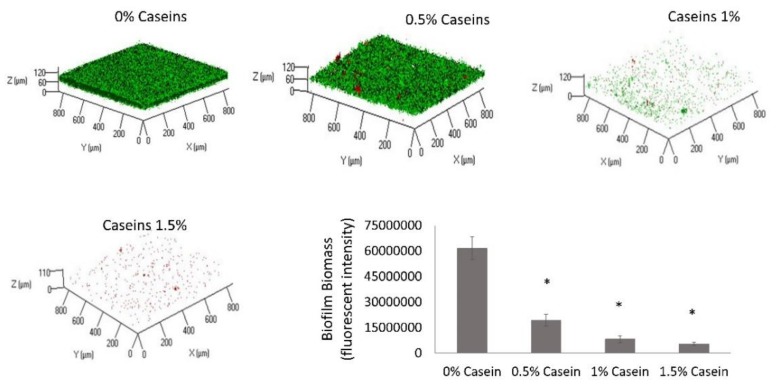
Supplementation of protease inhibitors eliminates proteolytic effect of *B. subtilis* cells in the presence of casein proteins. *S. mutans* and *B. subtilis* cells were grown in BHI supplemented with 3% lactose with various concentrations of casein proteins mixture (0%, 0.5%, 1%, or 1.5%) with addition of a proteinase inhibitor cocktail. After 24 h of incubation at 37 °C in 95% air/5% CO2, the formed biofilms were washed twice. The formed biofilms were stained in Syto 9 (green) labeling live bacteria and PI (red) labeling dead bacteria. The graph displays data of fluorescent integrity of the formed biofilms as calculated by imageJ. The protease inhibitor cocktail canceled the proteolytic effect of *B. subtilis* in the dual-species culture, and there was a significant decrease in the formed biofilm (in all tested concentrations of casein proteins). The pictures are representative of two biological repeats, each preformed in duplicate. * *p* < 0.05 compared to control (0% casein).

We finally wished to confirm whether the proteolysis ability of *B. subtilis* represents a major factor that allows the biofilm formation by *S. mutans* in the presence of caseins. It was found that there was a significant decrease in the formed biofilm in the dual-species culture (regardless of the tested concentration of casein proteins) when the protease inhibitor cocktail was added to the medium (Figure 6).

## 4. Discussion

Dental biofilm formation, which is highly dependent on nutrient and food consumption, represents a prerequisite for the majority of dental disorders [6,11]. Therefore, several studies investigated the role of milk and its products in biofilm formation, especially biofilm formed by *S. mutans*. Nonetheless, the effect of milk components on biofilm formation by oral pathogens is not explicit in many cases. Current thinking in the field is that certain milk proteins may negatively affect biofilm formation. Indeed, our results stand in line with previous studies, which showed that certain casein proteins are capable of decreasing biofilm formation in a dose-dependent manner [13,14,15]. 

However, not only milk constituents may affect biofilm formation; commensal bacteria that exist in dairy products may also do so [29]. For example, it was suggested that *Lactobacillus reuteri*, which can be found in yogurt, has an inhibitory effect on *S. mutans*, while other strains of lactobacilli from various kinds of yogurts did not show the same effect [29]. Therefore, it was of interest to examine the influence of other foodborne bacteria, which are associated with dairy products, on *S. mutans* biofilm formation. Milk and its products are subjected to pasteurization processes, which may not eradicate all of the existing bacteria. Spore-forming *Bacillus* can survive these processes and, thus, may interact with and affect the biofilm formation by oral bacteria [30,31].

Thus, we investigated how the presence of *Bacillus* species would affect the milk caseins on the surface attachment and biofilm formation by *S. mutans*. Characterization of biofilms formed in the dual-species culture, in the presence of caseins, showed no significant reduction in the biofilm biomass compared to control. Testing the ratio between the bacteria in the dual-species culture indicated that most of the cells in the mutual biofilm belong to *S. mutans*, and the number of *B. subtilis* cells was relatively low. A previous study showed that the initial interaction between *B. subtilis* cells and *S. mutans* during dual biofilm formation is related to dextran-associated EPS formed by *S. mutans* [20]. However, the biofilm in this model was formed in the presence of lactose, the main sugar in milk. In the presence of lactose, the EPS profile by *S. mutans* is different than the EPS formed with sucrose, and dextran is not dominant [12]. This might explain a reduction in the amount of *B. subtilis* DNA in the dual-culture biofilm. In high concentrations of casein proteins, there is an increase in the amount of DNA, which might be due to environmental DNA (eDNA), which could account for the increase in dead cells [32]. 

Since the type of surface is important and may affect the adherence and initial binding of the cells, we tested the same condition on hydroxyapatite discs, which resemble tooth enamel. SEM images showed that casein inhibits the adhesion of *S. mutans* to hydroxyapatite discs at 2%, similar to the results shown by Vacca-Smith and Bowen in 2000 [13]. However, when *B. subtilis* was present in the medium, *S. mutans* cells adhered to the hydroxyapatite surface. However, unlike the polystyrene surface, at the hydroxyapatite disc, there was still some reduction in the formed biofilm. A conceivable explanation for this could be a possible absorption of casein proteins onto hydroxyapatite, especially as a result of the decreasing pH [33], owing to the *S. mutans* metabolism of lactose [12]. Moreover, there can be a time limit of the breakdown of caseins by *B. subtilis*, which meanwhile allow a fast absorption of casein onto HA [14].

Most of the studies conducted concerning milk and its effect on oral biofilm formation attributed the findings to the interaction of the casein proteins, mainly κ-casein, which adhere to the adhesion-like protein in the *S. mutans* cell surface and inhibit its binding to the enamel surface [14,15]. On the other hand, it was shown that enzymatic activity reduces the efficacy of dairy powders to inhibit *S. mutans* adherence to HA [34]. Furthermore, our results suggested that, while *S. mutans* lacks the ability of milk protein proteolysis, *B. subtilis* could efficiently hydrolyze casein proteins, particularly κ-casein. Our results indicate that *B. subtilis* cells produce many enzymes that can breakdown casein proteins (including κ-casein), including both intracellular (as demonstrated by the SDS-PAGE) and extracellular enzymes (as indicated by the casein zymogram). Therefore, we speculate that the *B. subtilis* ability to hydrolyze caseins by its proteases leads to a breakdown of the casein proteins, which inactivates their action as adhesion-like proteins, thus inhibiting the binding to the surface. Indeed, the introduction of protease inhibitors to the media resulted in a notable decrease in the formed biofilm in the dual-species culture. The addition of the protease inhibitors abolished the proteolytic effect of *B. subtilis* cells, which contributed to the formation of the *S. mutans* biofilm in the presence of the casein proteins. 

## 5. Conclusions

**Figure 7:**
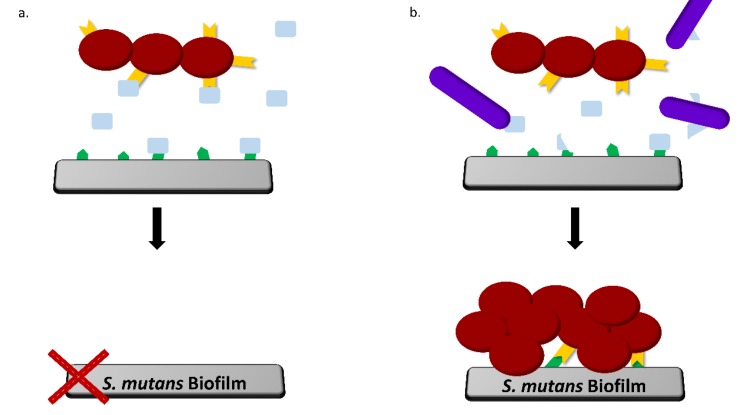
*B. subtilis* enables *S. mutans* adherence and biofilm formation due to its proteolytic activity. A suggested mechanism: (**a**) in the *S. mutans* mono-species culture, certain receptor molecules on the surface (green) and the bacterial surfaces (yellow) are blocked by the caseins (pale blue). (**b**) In the dual-species culture, *Bacillus* spp. cells (purple) can break down casein proteins, particularly κ-casein. The proteolysis decreases the interaction with the receptors and enables *S. mutans* to adhere to the surface and form a biofilm.

Overall, the results of this study challenge a notion regarding the “caries-safe” effect of milk. While casein proteins are capable of decreasing biofilm formation by *S. mutans*, commensal bacterial species that are found in milk may break down certain casein proteins in such a way that it becomes non-effective in the prevention of initial adhesion of oral bacteria (Figure 7).

## Supplementary Materials

The following are available online at https://www.mdpi.com/2076-2607/8/2/221/s1.
