# Supplementary material for: Proteolytic Activity of Bacillus subtilis upon κ-Casein Undermines Its “Caries-Safe” Effect
Source: Microorganisms. 2020 Feb 6;8(2):221. doi: 10.3390/microorganisms8020221 (PMC7074799; doi:10.3390/microorganisms8020221)
Supplement: Supplementary file 1 [file microorganisms-08-00221-s001.pdf]

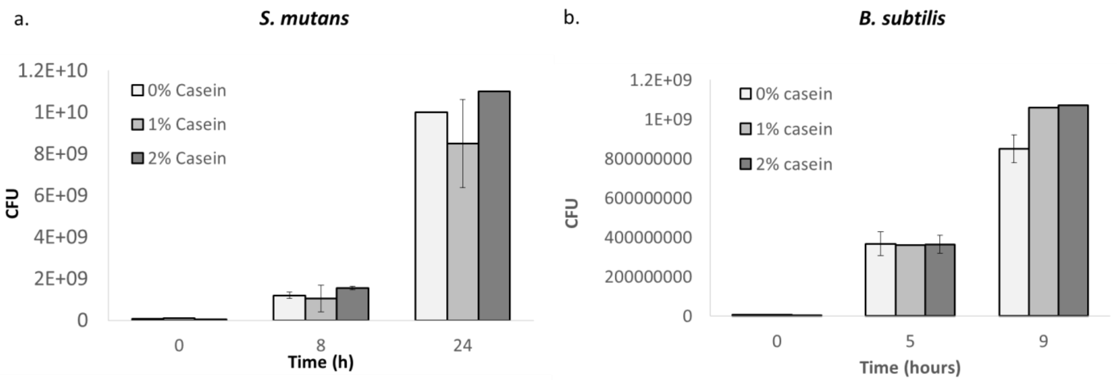

Figure S1. Growth curve analysis in the presence of milk caseins. (**a**) *S. mutans* cells were grown in BHI supplemented with various concentrations of the casein proteins mixture (0, 1 or 2%) and incubated at 37 °C in 95% air/5% CO_2. At each time point, a sample was taken, and CFU quantification was conducted. (**b**) *B. subtilis* cells were grown in LB supplemented with various concentrations of the casein proteins (0, 1, or 2%) and incubated at 37 °C, 150 rpm. At each time point, a sample was taken, and CFU quantitation was conducted. The data display a mean of 2 biological repeats, each performed in triplicate.
